# Graduate Study on the Continent—Germany

**Published:** 1908-05-30

**Authors:** 


### HOSPITAL ADMINISTRATION.

### CONSTRUCTION AND ECONOMICS.

### GRADUATE STUDY ON THE CONTINENT—GERMANY.

IV.—A "FERIEN KURSUS."

Ferien Kursen are vacation courses given in the interests of the general practitioner by specialists and professors attached to the various clinics at the German and Austrian universities. They are, perhaps, the ideal of graduate courses, fulfilling, as they do, the objects which should be the chief concern of the promoters of graduate study. That is to say, they give the men the chance of perfecting their knowledge in general medicine and in special subjects, an opportunity of benefiting by the large amount of clinical material available at the general hospitals, and finally the means of becoming practically acquainted with new methods and the most recent advances in diagnosis and treatment. The development of this branch of graduate study has indeed been remarkable in Germany, where the "practising physician" has been given opportunities which are quite beyond his reach in England. In Berlin, for instance, no fewer than sixty-two courses were announced during the present March vacation—all by men of standing and eminence in the profession, and the majority "free," or to be attended on payment of an honorarium which is in most cases merely nominal. The foreign graduate is not entitled to attend these courses, at least those which are free, but an interview with the professor is usually sufficient to secure an invitation to attend, and no difference whatever is made between foreign and native practitioners who attend the course.

Courses are given in nearly every imaginable subject—in diagnosis, treatment, ætiology, and pathology of general medicine and surgery, in special branches such as rhinoscopy, radioscopy, "instrumenten lehre" (which means the demonstration of new and old instruments and appliances, a very useful and instructive course), cystoscopy, nervous diseases, orthopædics, special surgery, therapeutics, balneology, tropical medicine, medical jurisprudence, psychiatry, etc. Full details about every course are usually published a month before the vacation in most of the leading medical journals, or are posted at the offices of the various Post-graduate Associations, such as those mentioned in my last article. As an illustration of the kind of teaching, I cannot do better than describe one of the recent courses at the Berlin Charité, given by the Professor of the Surgical Polyclinic, Dr. Pels-Lensden.

This course consisted of two divisions—practical operative work on the cadaver and on the dog, daily from half-past seven to half-past nine in the morning; and surgical diagnosis and treatment on three afternoons a week—and was one of several similar courses given during the vacation. It was limited to eight members, who were all practising physicians—general practitioners who had taken a holiday to run up to Berlin for a month's study. All were keen, critical, and eager for work, and they got the utmost out of their month's special study. Early in the morning the operative class began its work, practising at first minor operations and going on to major work. There was material enough—two men on a "body" and a fresh "subject" every week. Excisions, amputations, resections, which in an ordinary hospital operative class the student has only an opportunity of practising once, were done again and again, different methods being practised, though, as is perfectly right, the greatest stress was laid upon "knowing one method perfectly." The operations on dogs were done with the same care and scrupulous attention to asepsis and after-treatment which would have been insisted upon in the case of patients. They were chiefly intestinal operations, and gave the men an exceptional opportunity of seeing for themselves the difficulties encountered in such operations. Whether they taught much more is questionable, for a gastro-enterostomy on a normal dog is, after all, perfectly different from the same operation performed on a human patient under pathological conditions.

Twice a week—on Tuesdays, Thursdays, and Fridays, from two to four o'clock in the afternoon—surgical diagnosis and treatment were discussed, patients being shown and cases demonstrated. The class met in the large room at the Polyclinic, and from eight to twelve different cases were shown. All were interesting, not, perhaps, because they presented rare lesions or out-of-the-way conditions (as a matter of fact, none were really museum cases), but because they were excellent cases for demonstration purposes, representing conditions such as are likely to be met with in general practice. The patient was shown, examined by the class, and the diagnosis and treatment were fully discussed, in many cases the pathology and morbid anatomy of the lesions being discussed as well. Where the case demanded an operation such as could be done under local anæsthesia or treatment such as could be applied to out-patients, the class was asked to do such operation or apply such treatment under the Professor's supervision.

The men are taught what they can apply in practice, not elaborate methods which need assistance and a large armamentarium. The value of the course it would be difficult to overestimate, for during the month each man sees between 120 and 150 different cases, and does a dozen or more "minor" operations, such as a fortunate dresser may be expected to do during his three months' term of office at our large hospitals. The men criticise and discuss freely, and the professor is always ready to meet difficulties and to explain matters. The relations existing between the class and the demonstrator are excellent. The latter knows that he is not dealing with students, and the men are able to bring to their work practical experience and knowledge which make the task of diagnosis easier. To some these small operations may seem to be *infra dig.* for the qualified practioner to do in a class. None of the men appeared to think it beneath them to do the most minor of operations, and it was easy to see that they learned by doing it, learned to use the injecting syringe, the methods of local anæsthesia, and to be thorough and exact.

Nor do the other courses differ from this surgical one. Those men I have spoken to who have attended the medical courses are loud in their praise of the value of the work done. Everyone acknowledges that attendance at a vacation course is not time wasted but, on the contrary, time very worthily expended in learning something new and gaining practical experience. To a large extent the success of these vacation courses is due to the self-sacrificing zeal of the professors and demonstrators, who really do their utmost to help their classes. It would be excellent were such a system introduced with us, whereby our best clinical teachers could give a series of free lectures or demonstrations, analogous to these courses, to qualified general practitioners.

Such a series of lectures would be well attended, and if they are properly arranged they would be of incalculable benefit. A leaf out of the book of the German post-graduate associations would be useful to our associations; but before we can expect to reach the heights which are here attained, we shall have to stimulate the general interest of the profession—and especially of the leaders of the profession—in graduate study. For without the cordial co-operation of the leaders of the profession here—a co-operation which necessarily demands a certain amount of self-sacrifice—the Ferien Kursus in Germany would not be the unqualified success that it is.

